# Checkpoint kinase 1 expression is an adverse prognostic marker and therapeutic target in MYC-driven medulloblastoma

**DOI:** 10.18632/oncotarget.10692

**Published:** 2016-07-19

**Authors:** Eric W. Prince, Ilango Balakrishnan, Monil Shah, Jean M. Mulcahy Levy, Andrea M. Griesinger, Irina Alimova, Peter S. Harris, Diane K. Birks, Andrew M. Donson, Nathan Davidson, Marc Remke, Michael D. Taylor, Michael H. Handler, Nicholas K. Foreman, Sujatha Venkataraman, Rajeev Vibhakar

**Affiliations:** ^1^ Department of Pediatrics and Section of Pediatric Hematology/Oncology/BMT, Children's Hospital Colorado and University of Colorado Denver, Anschutz Medical Campus, Aurora, CO, United States; ^2^ University of Colorado School of Medicine, Aurora, CO, United States; ^3^ Division of Pediatric Neurosurgery, Children's Hospital Colorado and University of Colorado Denver, Anschutz Medical Campus, Aurora, CO, United States; ^4^ Division of Neurosurgery, Program in Developmental and Stem Cell Biology, Hospital for Sick Children, Toronto, ON, Canada; ^5^ DKFZ German Cancer Research Center, University Hospital Düsseldorf, Heidelberg, Germany

**Keywords:** medulloblastoma, CHK1, Myc

## Abstract

Checkpoint kinase 1 (CHK1) is an integral component of the cell cycle as well as the DNA Damage Response (DDR) pathway. Previous work has demonstrated the effectiveness of inhibiting CHK1 with small-molecule inhibitors, but the role of CHK1 mediated DDR in medulloblastoma is unknown. CHK1, both at the mRNA and protein level, is highly expressed in medulloblastoma and elevated CHK1 expression in Group3 medulloblastoma is an adverse prognostic marker. CHK1 inhibition with the small-molecule drug AZD7762, results in decreased cell growth, increased DNA damage and cell apoptosis. Furthermore, AZD7762 acts in synergy with cisplatin in reducing cell proliferation in medulloblastoma. Similar phenotypic changes were observed with another CHK1 inhibitor, PF477736, as well as genetic knockdown using siRNA against CHK1. Treatments with small-molecule inhibitors of CHK1 profoundly modulated the expression of both upstream and downstream target proteins within the CHK1 signaling pathways. This suggests the presence of a feedback loop in activating CHK1. Overall, our results demonstrate that small-molecule inhibition of CHK1 in combination with, cisplatin, is more advantageous than either treatment alone, especially for Group 3 medulloblastoma, and therefore this combined therapeutic approach serves as an avenue for further investigation.

## INTRODUCTION

Medulloblastoma is the most common malignant brain tumor of childhood [1]. Recent genomic investigation has identified four subgroups of medulloblastoma with variable clinical outcomes, illustrating genetic heterogeneity of medulloblastoma and emphasizing the need for new therapeutic interventions based on the molecular signatures of these subgroups [2–4]. Although various signaling pathways (such as Sonic Hedgehog and Wnt) have been associated with medulloblastoma cell biology, novel therapeutic interventions based on this knowledge have seen limited development [5, 6]. Current mainstay medulloblastoma therapies (surgical resection, craniospinal irradiation, and chemotherapy) have yielded reduced mortality in average-risk patients, however in high-risk patient outcomes remain suboptimal [7, 8]. As chemotherapy and craniospinal radiation both have significant long-term irreversible side effects, there is clear indication for the development of a novel medulloblastoma treatment [9–13].

Protein kinases play a key role in cell cycle regulation and proliferation making them an ideal target for cancer therapy. In order to explore new kinase targets, we utilized transcriptional profiling for medulloblastoma samples, as well as a kinome-wide siRNA screen, identifying eight kinases that play central roles in tumor cell viability [14]. Three of these kinases (Aurora Kinase A, Polo-Like Kinase A, and WEE1) have been demonstrated to exhibit therapeutic value in data presented previously by ourselves and others [14–18]. Additional analysis of our previous data, along with the aforementioned microarray and siRNA screen results, indicate Checkpoint Kinase 1 (CHK1) as a differentially-expressed potential therapeutic target in medulloblastoma.

To ensure cellular survival, it is paramount that cells have the ability to regulate homeostasis and prevent neoplastic conversion. In order to maintain cellular viability, there exists a complex series of critical surveillance and repair mechanisms. One such mechanism is known as the DNA Damage Response (DDR) pathway which, in response to DNA damage, rapidly induces cell cycle arrest, DNA damage repair mechanisms, and – if necessary – apoptosis, via recruitment of the ATM/ATR kinase pathway. Upon activation of ATM/ATR the downstream target CHK1 (a serine-threonine kinase responsible for the regulation of the G_2_/M cell cycle checkpoint) becomes phosphorylated and transiently halts the cell cycle progression to allow for DNA damage repair [19–24]. Although cell cycle arrest and DNA repair mechanisms protect integrity of normal cells, it may also diminish the effectiveness of cancer therapy by facilitating resistance in tumor cells. Furthermore, a majority of the tumors are deficient in the G_1_ DNA damage checkpoint pathway and other components of checkpoint signaling, making them solely reliant on the G_2_/M checkpoint for DNA repair [19, 21]. Thus, abrogation of G_2_/M checkpoint via the inhibition of CHK1 activity could provide an optimal therapeutic route to selectively sensitize tumor cells to chemotherapy and remove subsequent resistance mechanisms. Recently, studies combining CHK1 inhibitors with a cytotoxic agent have shown promising results in adult and pediatric solid tumors, however, the role of inhibiting CHK1 in the context of medulloblastoma is not well characterized [22, 25].

Reported herein, we sought to evaluate the role of CHK1 as a potential therapeutic target in medulloblastoma. We first confirmed differential expression of CHK1 in samples from two independent medulloblastoma patient cohorts. Next, we examined the effects of inhibiting CHK1 activity in terms of tumor cell viability, cell colony formation, and relative apoptosis. Finally, we examined the impact of inhibiting CHK1 in combination with the standard-of-care therapeutic compound cisplatin in medulloblastoma cells *in vitro* using the small molecule inhibitor AZD7762.

## RESULTS

### CHK1 is overexpressed in medulloblastoma

**Figure 1:**
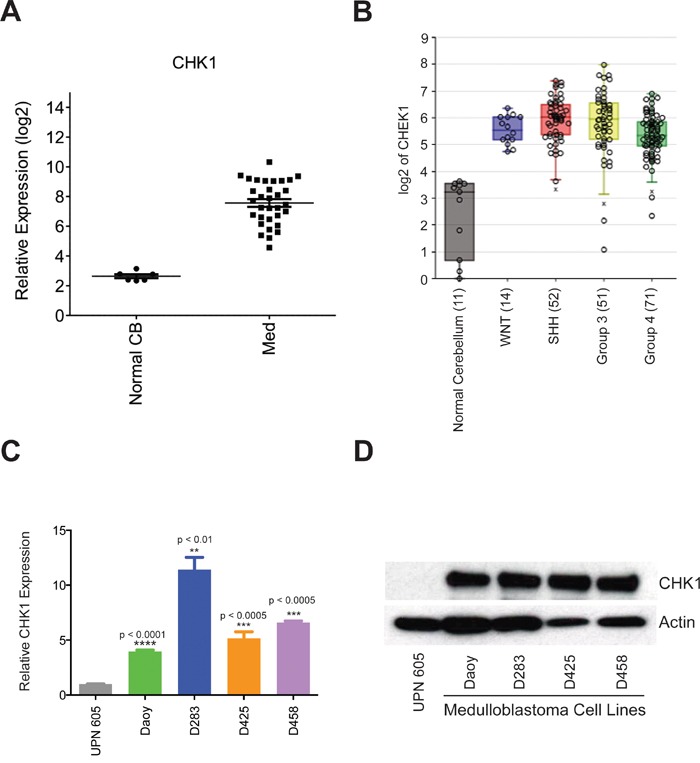
CHK1 is overexpressed in medulloblatoma **A.** CHK1 expression is found to be elevated in medulloblastoma patient samples when compared to normal cerebellum in a cohort of 16 samples. **B.** CHK1 expression is elevated in all subgroups of medulloblastoma patient samples when compared to normal cerebellum in a cohort of 90 samples. **C.** CHK1 mRNA is elevated in medulloblastoma tumor cell models compared to normal cerebellum (UPN 605) when analyzed via qRT-PCR. **D.** CHK1 protein expression is increased in medulloblastoma tumor cell models in comparison to normal cerebellum when analyzed via western blot.

Through genomic analysis, we recently demonstrated that multiple kinases regulating the G_2_/M cell cycle check point are key mediators of medulloblastoma cell viability. Among the kinases we identified was CHK1. We hypothesized that a kinase regulator of DNA damage during the cell cycle would be an ideal candidate as a therapeutic target in medulloblastoma. To test our hypothesis, we analyzed CHK1 mRNA expression in a cohort of 16 medulloblastoma patient samples. We found the expression of CHK1 mRNA was high in all patient samples when compared to normal cerebellum (Figure 1A). Analyses have defined four major subgroups of medulloblastoma which are Sonic Hedgehog Signaling (SHH), Wnt signaling (WNT), Group 3 (MYC-amplified), and Group 4 [2]. To further elucidate whether there was a correlation within the subgroups of medulloblastoma, we examined expression of CHK1 mRNA in second cohort (n=120) of medulloblastoma patient samples. When examined at the genomic subgroup level, there was no difference in CHK1 mRNA expression among genomic subgroups but all four groups showed increased levels of CHK1 compared to normal cerebellum. (Figure 1B). It is possible that CHK1 expression is decreased in normal cerebellum due to decreased numbers of proliferating cells. To address this we compared CHK1 levels in murine P5 cerebella to group 3 medulloblastoma cell lines. We found that CHK 1 expression is significantly higher in proliferating tumor cell lines compared to proliferating normal cerebellum (Supplementary Figure S1) These data indicate that CHK1 mRNA up-regulation may not be specific to a molecular subgroup of medulloblastoma. We further evaluated the expression of CHK1 mRNA in a panel of well-characterized medulloblastoma cell lines. All medulloblastoma cell lines expressed significantly higher levels of CHK1 when compared to pediatric normal cerebellum, which was consistent with our patient tissue sample data (Figure 1C). We next examined CHK1 protein expression in normal pediatric cerebellum as well as a panel of medulloblastoma cell lines. Compared to normal pediatric cerebellum, CHK1 protein expression was increased in all medulloblastoma cell lines (Figure 1D).

**Figure 2:**
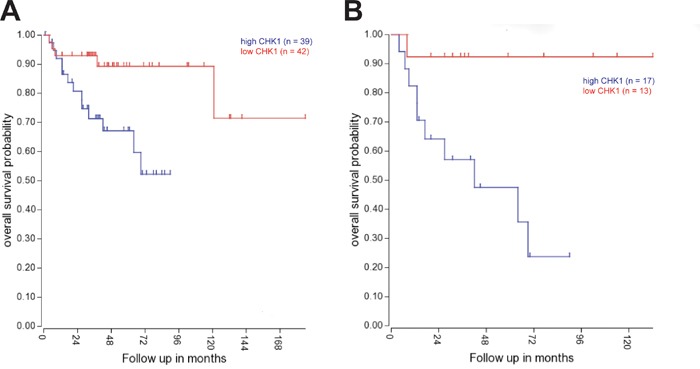
CHK1 expression is correlated with adverse outcomes in medulloblastoma patients **A.** Kaplan-Meyer survival curve shows a decrease in long-term survival of medulloblastoma patients with increased CHK1 expression across all four sub-groups. **B.** Kaplan-Meyer survival curve shows significant decrease in long-term survival of medulloblastoma patients with increased CHK1 expression in group 3 tumors (p < 0.01).

We next decided to examine the impact of CHK1 expression on patient survival. Gene expression and survival data from 204 patients (Pomeroy data set) was evaluated using R2 microarray analysis and visualization platform (http://r2.amc.nl). High CHK1 expression is associated with adverse outcomes in medulloblastoma over all (Figure 2A). Subgroup analysis shows that CHK1 expression is particularly predictive of poor outcome in Group 3 tumors (Figure 2B, p < 0.01) but not SHH or Group 4 tumors (Supplementary Figure S2).

### AZD7762, a small molecular inhibitor of CHK1, potently suppresses medulloblastoma cell growth *in vitro*

**Figure 3:**
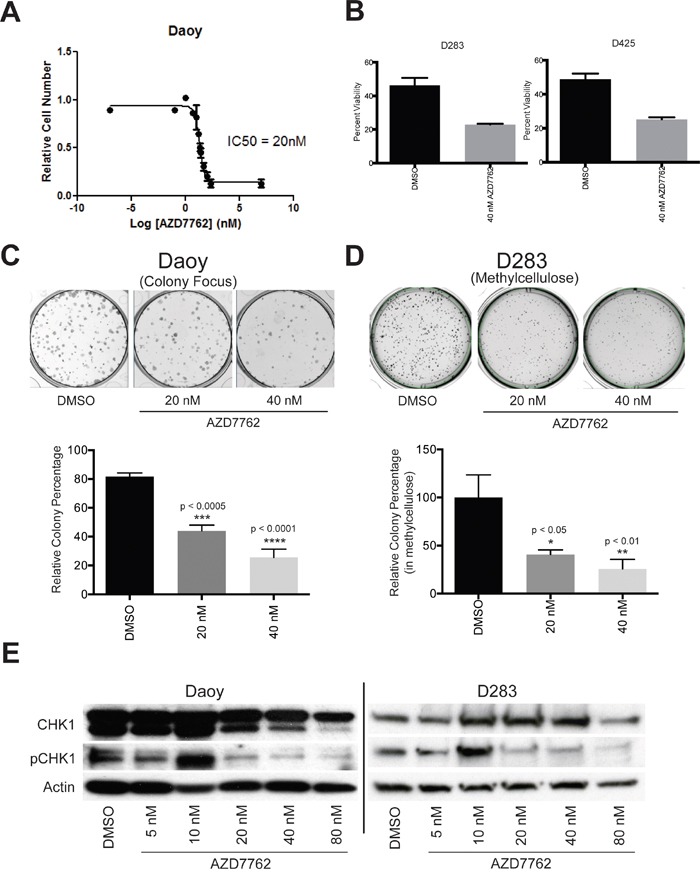
Small-molecule CHK1 inhibitor, AZD7762, potently suppressed medulloblastoma growth *in vitro* **A.** IC_50_ concentration of AZD7762 (20 nM) in Daoy cells was determined using MTS absorbance data and nonlinear regression analysis. **B.** cellular viability is significantly decreased in D283 (left) and D425 (right) tumor cells when treated with AZD7762. **C.** The capacity for colony formation was significantly decreased in Daoy (left) and D283 (right) medulloblastoma tumor cell models when treated with AZD7762. **D.** phosphorylated-CHK1 protein expression is decreased in Daoy (left) and D283 (right) tumor cells relative to vehicle control.

Several CHK1 inhibitors have been recently described as potentially possessing therapeutic utility [24]. AZD7762, a thiophene carboxamide urea, is one of these inhibitors [26]. We examined the effect of CHK1 inhibition using AZD7762 on proliferation of medulloblastoma cells. Daoy cells were treated with varyious concentrations of AZD7762 and cell proliferation was measured by MTS assay. AZD7762 was a potent inhibitor of Daoy cell growth with IC_50_ of 20 nM (Figure 3A).

To further characterize chemical inhibition of CHK1 we examined the impact on viability of 2 well characterized medulloblastoma cell lines that carry Myc translocations and are part of the Group 3 genomic sub-group. Cell viability was assessed following AZD7762 drug treatment using Guava EasyCyte Plus flow cytometer. D283 and D425 cells were treated with 40nM AZD7762 for 72 hours. After treatment, cell viability decreased with 40 nM AZD7762 in both D283 and D425 cell lines (Figure 3B).

To evaluate the impact of AZD7762 on long-term proliferation, we performed colony formation and methylcellulose assays on Daoy and D283 cell lines, respectively. In Daoy tumor cells, 72-hour exposure to 20 nM and 40 nM AZD7762 significantly decreased colony number (Figure 3C, p < 0.0005). Similarly, AZD7762 treatment significantly suppressed the ability to form colonies in D283 cells (Figure 3D, p < 0.05) in methylcellulose. These data reflect the impact of CHK1 inhibition on cell viability of medulloblastoma cells.

In order to assess whether AZD7762 was inhibiting CHK1 activity, we measured the protein expression levels of phosphorylated CHK1 by immunoblotting. A decrease in levels of phosphorylated-CHK1 was seen in a dose-dependent manner with a range of AZD7762 treatment in Daoy and D283 cell lines (Figure 3E). The levels of CHK1 showed no significant change with varying concentration of AZD7762 treatment as anticipated.

### Treatment with AZD7762 induces DNA damage

**Figure 4:**
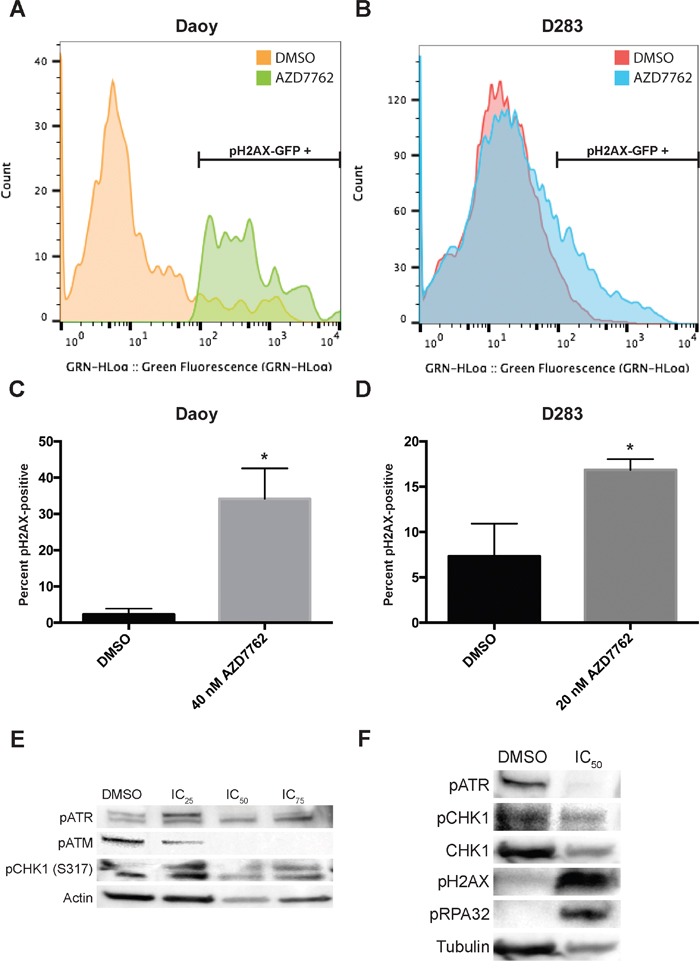
Treatment of medulloblastoma tumor cells with AZD7762 induces DNA damage **A-D.** Daoy (A, C) and D283 (B, D) tumor cells were treated for 72 hours with AZD7762 and incubated with a γH2AX-GFP flow cytometry flourophore for analysis. Representative GFP emission profiles are shown (A, B) as well as quantitative data for treatment versus DMSO vehicle control (C, D, p > 0.05). **E.** treatment of Daoy cells with AZD7762 revealed a decrease in pATR and pCHK1 protein expression as well as an increase in pH2AX and pRPA32 protein expression. **F.** treatment of Daoy cells with PF477736 revealed a decrease in pATM and pCHK1 protein expression.

DNA damage repair pathways play a critical role in survival of tumor cells, especially following treatment with DNA damaging agents. γH2AX is recruited to sites of DNA damage and is removed after DNA repair. To assess the impact of AZD7762 on medulloblastoma cells, we analyzed DNA damage following treatment with AZD7762 by staining with an anti-γH2AX flourophore antibody and subsequently measured via flow cytometry. Results analyzed using flow cytometric software reflected increased DNA damage as measured by γH2AX foci in Daoy and D283 cell lines when treated for 72 hours with 40 nM and 20 nM of AZD7762, respectively (Figure 4A). Quantitative analysis of the data showed DNA damage was significant when compared to DMSO control for both Daoy (p < 0.01) and D283 (p < 0.05) cell lines (Figure 4B).

To examine more closely the impact of CHK1 inhibition on DDR proteins we evaluated levels of phosphorylated ATR and ATM and phosphorylated CHK1 in medulloblastoma cells. When medulloblastoma cells, Daoy treated with varying concentrations of AZD7762, there is a decrease in pCHK1, pATM and no significant changes in pATR (Figure 4E). Similarly when these cells were treated with another CHK1 inhibitor, PF477736, there is a drastic decrease in pATR, pATM and pCHK1 (Figure 4F). Like wise there is a significant induction of phosphorylation of pH2AX and pRPA32 suggestive of induction of DNA damage (Figure 4F). We also evaluated phosphorylation of associated proteins CHK2 and p53. AZD7762 did not alter phosphorylation of CHK2 but PF477736 did decrease CHK2 phosphorylation indicating a less specific mechanism for this drug. Both compounds induced phosphorylation of p53 as expected (Supplementary Figure S3).

### AZD7762 induces apoptosis in medulloblastoma cells

**Figure 5:**
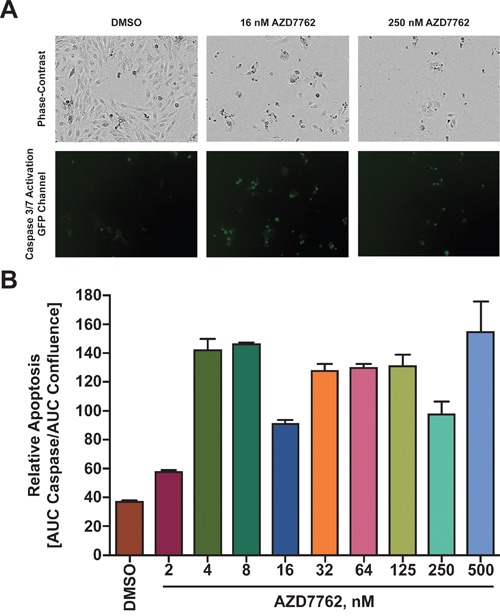
Treatment of Daoy cells with AZD7762 induces apoptosis **A.** Daoy cells transduced with a Caspase responsive GFP reporter were seeded and 24 hours later treated with varying concentrations of AZD7762. Cells were monitored in bright-field and green fluorescent channels with representative images shown. **B.** Apoptosis was quantized by dividing the integral of the green fluorescent emission profile (effectively caspase 3/7 expression) over the integral of the phase-contrast emission profile (effectively total cell number).

Treatment of medulloblastoma cells with AZD7762 significantly decreased cell proliferation. In order to determine if this effect was due to apoptosis, an IncucyteZOOM was used to kinetically measure caspase-3/7 mediated apoptosis. Stimulation of intrinsic or extrinsic apoptotic pathways triggers a signaling cascade resulting in activation of caspase-3 that can serve as an apoptotic signaling marker. Cells were treated with varying concentration of AZD7762 for a 72-hour period and caspase activation measured by a caspase-3/7 GFP reporter and change in cell density was measured every four hours. Cumulative caspase activation over the 72 hours was obtained by calculating the area under the curve (AUC). This was normalized to cell density over time calculated by the AUC of confluence over the 72-hour period. In comparison to DMSO, a significant increase in apoptosis was observed in Daoy cells treated with 16 nM and 250 nM AZD7762 drug for 72 hours which was visualized (Figure 5A) and quantified (Figure 5B). Furthermore, increase in cellular apoptosis correlated directly with increasing concentration of AZD7762 (Supplementary Figure S4).

### AZD7762 acts in synergy with cisplatin

**Figure 6:**
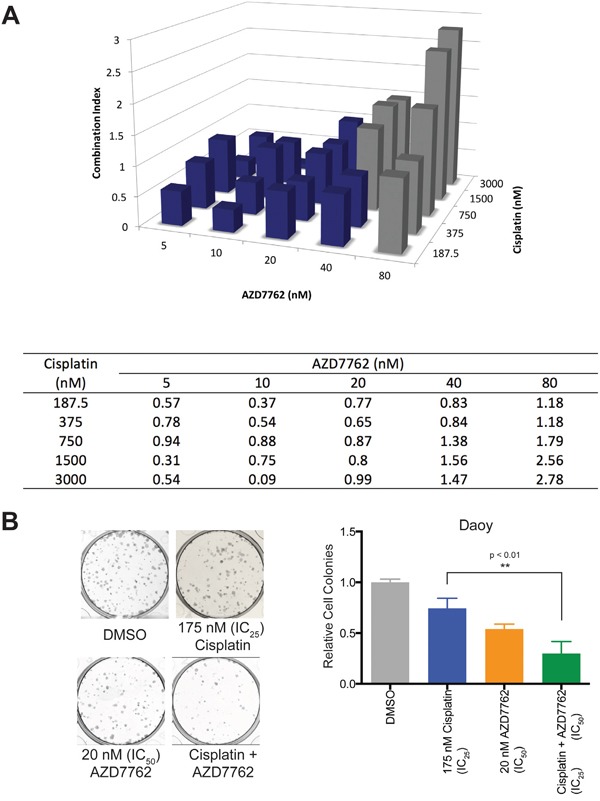
Treatment of Daoy cells with AZD7762 acts in synergy with cisplatin to reduce cellular proliferation **A.** Daoy cells were treated in triplicate with varying combinations of AZD7762 and cisplatin for 72 hours and cellular proliferation was analyzed using an MTS assay. Values were computed using the Chou-Talalay Combination Index (CI) Theorem; Three-dimensional bar graph (top) and a table of values (bottom) are shown. Grey boxes are presumed to contain dead cells and are omitted in consideration of synergy. **B.** Using CI values, colony formation was studied using 20 nM AZD7762 and/or 175 nM cisplatin with representative well images (left) and quantitated cell colony count data shown (right).

Most chemotherapy protocols for medulloblastoma involve cisplatin. Cisplatin use has significant adverse effects including ototoxicity, nephrotoxicity, and severe bone marrow suppression. Thus, drugs that work synergistically will allow reduction in the dose of standard chemotherapeutic agents. We examined the ability of AZD7762 to act in synergy with cisplatin using an MTS assay. Based on the optical densities measured, combination-index values were calculated in Daoy cell lines using the CIE method as described in the methods section. Combination index values showed synergy in reducing cellular proliferation of Daoy cells between AZD7762 and cisplatin at varying concentrations below the IC_50_ value of AZD7762 (Figure 6A).

To further assess the long-term impact of combination therapy on medulloblastoma cells, we used the colony formation assay. Daoy cells were exposed to AZD7762, cisplatin, or combination of both for 72 hours and colony formation was assessed as described in methods section. Compared to AZD7762 or cisplatin alone, Daoy cell line had significantly lower number (p < 0.01) of colonies formed in combination treatment group. (Figure 6B). Representative colony wells with and without treatment were shown.

### CHK1 inhibition overall reduces cellular viability in medulloblastoma cells

**Figure 7:**
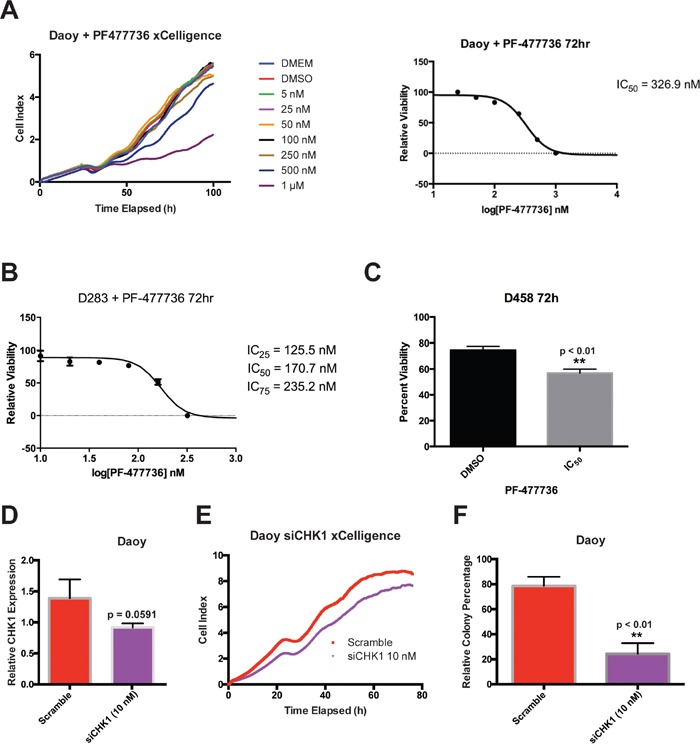
CHK1 inhibition via small-molecular inhibitor PF477736 and siCHK1 knockdown reduce cellular viability in medulloblastoma cells **A.** Treatment of Daoy cells with PF477736 reduced cell proliferation in xCelligence assay (left) yielding an IC_50_ concentration of roughly 323 nM (right). **B.** 72 hour treatment of D283 cells with PF477736 reduced cellular viability yielding an IC_50_ value of roughly 170 nM. **C.** 72 hour treatment of D458 cells with PF477736 showed a significant reduction in cellular viability (p < 0.001). **D-F.** Transfection of Daoy cells with siCHK1 was confirmed to reduce CHK1 expression via qRT-PCR (p = 0.0591, left), reduced cellular proliferation via xCelligence (center), and significantly decreased colony formation (p < 0.001, right).

We sought to investigate if the results presented above were a product of AZD7762 treatment specifically or a result of CHK1 inhibition. To do this we first treated Daoy, D283, and D458 medulloblastoma cells with an alternative small-molecule CHK1 inhibitor, PF477736 [27]. Similar to AZD7762, we found a decrease in cellular proliferation using xCelligence assay in Daoy (Figure 7A) and a flow cytometric cell viability assay D283 (Figure 7B) cells as well as a significant decrease in cellular viability in another medulloblastoma cell model, D458 (p < 0.005, Figure 7C).

To confirm whether genetic knockdown of CHK1 leads to similar phenotypic changes observed with small-molecule drugs, AZD7762 and PF477736, we performed an siCHK1 transfection in Daoy cells.

CHK1 knockdown was confirmed via qRT-PCR (Figure 7D) and was shown to decrease cellular proliferation (Figure 7E) and clonogenic assay in Daoy cells (Figure 7F). Taken together, these results suggest that our findings are not an anomaly of AZD7762 treatment and are in fact a product of CHK1 inhibition; thus further validating the use of CHK1 as a therapeutic target for medulloblastoma patients.

## DISCUSSION

Genomic heterogeneity of medulloblastoma has created challenges in improving outcomes using traditional approaches. In addition, significant therapy related morbidity in patients with medulloblastoma has resulted in an urgent need for novel therapeutic targets. Although recent genomic studies have begun to characterize the molecular signature of medulloblastoma subgroups [2–4], the clinical progress in terms of designing novel agents based on this knowledge has been limited. Recent analysis of protein kinase gene expression has revealed that multiple protein kinases including those involved in cell cycle checkpoint regulation, such as CHK1, were differentially expressed in medulloblastoma [14, 15, 17].

CHK1 is a serine-threonine kinase which plays a critical role in DNA repair, mitotic spindle checkpoint, and regulation of apoptosis [28–30]. Specifically, it is a critical component of DNA damage response pathway which ensures genomic stability. CHK1 expression is elevated in many adult and pediatric cancers, including triple-negative breast cancer, non-small cell lung cancer, and neuroblastoma [31]. While CHK1 mRNA expression is elevated in medulloblastoma, its role in pathogenesis is not completely understood.

In this study, we demonstrate that CHK1 mRNA is overexpressed in two independent cohorts of medulloblastoma patient samples in comparison to normal cerebellum. We also showed that CHK1 upregulation was not specific to any subgroups of medulloblastoma. However CHK1 upregulation in Group 3 medulloblastoma demonstrated a particularly poor prognosis, perhaps due to its role in mitigating Myc driven replication stress. Furthermore, we demonstrated that inhibition of CHK1 by small molecule inhibitors AZD7762 and PF477736 as well as genetic knockdown of CHK1 in medulloblastoma cell lines results in decreased proliferation both in short and long-term assays. Treatment with 20nM AZD7762 also induced DNA damage and apoptosis in medulloblastoma cell lines. These results are similar to the effect of AZD7762 in other cancers [32, 33].

There are several CHK1 inhibitors currently undergoing clinical trials including SCH900776 and LY2606368 in other types of cancer. However, there are currently no clinical studies of CHK1 inhibitors in medulloblastoma. Our data and those presented in literature provide strong indication for such studies [34]. Most importantly, our analysis revealed that elevated expression of CHK1 correlated with poor long-term survival in patients with Group 3 subgroup medulloblastoma. Group 3 tend to be more aggressive in comparison to other subgroups [2, 4]. These results in combination with the lack of differences in CHK1 expression amongst medulloblastoma subgroups suggests that CHK1 upregulation may play a direct role in overall survival of patients with medulloblastoma and thus makes it a critical target for clinical trials.

Liu Q et al., showed that CHK1 is regulated by two upstream PIK family kinases ATM and ATR and that plays an important role in the G2/M DNA damage checkpoints and tumor suppression [35]. ATR and ATM are activated within minutes of DNA damage and increase the phosphorylation of CHK1. This in turn can work in a feedback loop mechanism and save the cells from cell death. Our data in medulloblastoma confirm these previous studies and further suggest that these drugs can be a promising therapeutic addition in medulloblastoma. For example it has been shown that CHK1 is crucial for the proliferation of T-ALL cells by preventing ATM/Caspase-3 dependent cell death [36]. Sarmento et al, reported that in T-cell acute lymphoblastic leukemia, ATM/ATR activation dependent CHK1 phosphorylation is involved in activating the replication stress response [36]. In agreement with these findings, we found by protein analysis that inhibition of CHK1 by PF477736, increased the levels of the replication stress mark pRPA32 and the DNA damage marker γH2AX. This suggests replication stress induced DNA damage leads to cell apoptosis when CHK1 activity is inhibited. We then tested whether CHK1 inhibition can sensitize cells to other chemotherapeutic agents when those agents induce DNA damage and can lead to positive feedback in ATM/ATR pathway that rescue cells from cell killing.

Chemotherapy forms a vital backbone of medulloblastoma therapy. However, it results in significant therapy related morbidity. CHK1 is activated in response to DNA-damaging agents like chemotherapy or radiation. Following DNA damage, activated CHK1 phosphorylates cdc25c which prevents downstream activation of CDK1 resulting in cell cycle arrest at G_2_/M checkpoint [21]. The G_2_/M cell cycle checkpoint is critical to ensure genomic repair prior to mitosis. Thus, abrogation of this checkpoint can lead to premature mitotic entry and subsequent cell death [37, 38]. In this study, we showed that low nanomolar concentrations of AZD7762 sensitized medulloblastoma cells to cisplatin. Combination index values further reflected synergy between AZD7762 and cisplatin. Thus, our results suggest that AZD7762 effectively enhanced chemo-sensitivity in medulloblastoma cells *in vitro*.

In summary, our data reveals that CHK1 inhibition has a critical role in medulloblastoma therapy in addition to other cancers [39]. The next step will be to perform cerebellar xenografts studies in mice to show efficacy of CHK1 inhibition treatment *in vivo*.

## MATERIALS AND METHODS

### Cell lines, primary patient samples, and murine samples

Primary medulloblastoma patient samples (n=16) were obtained from Children's Hospital Colorado and were collected in accordance with local and federal human research protection guidelines and Institutional Review Board (IRB) regulations after obtaining informed consent. Normal cerebellar samples in Figure 1 (A, C, and D) were obtained from nonmalignant brain biopsies or autopsies at the Children's Hospital Colorado under IRB guidelines. Normal cerebellar samples UPN 605 was obtained from a 5-year old patient autopsy.

The Daoy and D283 medulloblastoma cell lines were purchased from American Type Cell Culture (Rockville, MD). D425 and D458 cell lines were kindly provided by Dr. Darell D. Bigner (Duke University Medical Center, NC). Cell lines were cultures in DMEM (Gibco, Carlsbad, CA) supplemented with 10% fetal bovine serum (Atlanta Biologicals, Lawrenceville, GA) and 100 U/mL penicillin-streptomycin (Gibco).

Postnatal day 5 (p5) mouse normal cerebellum tissue samples were harvested in accordance with the University of Colorado IACUC established protocol.

### Transfection of RNAi vectors

Using the manufacturer's suggested protocol for reverse transcription, an siRNA (Silencer Select, Ambion) targeting CHK1 mRNA, siCHK1 (s503), and a non-targeting siRNA were transfected into Daoy cells using the siPORT NeoFX Transfection Agent (Ambion), with the final siRNA concentration at 10 nM transfected into the cells.

### Gene expression microarray analysis

Sixteen tumor samples from the first cohort were collected at the time of surgery and snap-frozen in liquid nitrogen. Tumors were homogenized and RNeasy kit (Qiagen, Valencia, CA) was used to extract ribonucleic acid from tumor samples which was hybridized to HG-U133 Plus 2.0 Gene-Chips (Affymetrix, Santa Clara, CA) to evaluate for gene expression. Microarray data from the samples was background-corrected and normalized using gcRMA algorithm. In subsequent analysis, one probe set per gene was used which was based on highest overall expression level across samples. Student's t-test was used to determine differential expression of genes [40].

Second cohort of 120 tumor specimens was obtained in accordance with the Research Ethics Board at the Hospital for Sick Children (Toronto, Canada). Gene expression array data were generated and analyzed as described [4].

### Group 3/4 survival data

Gene expression and survival data from 204 patients (Pomeroy data set) evaluated using R2 microarray analysis and visualization platform (http://r2.amc.nl).

### Quantitative real-time polymerase chain reaction

RNA was extracted from murine p5 normal and primary patient cerebellar tissue and primary via homogenization of tissue in RLT buffer supplemented with 10% 2-mercaptoethanol. mRNA from medulloblastoma cell lines, patient, and murine samples was isolated using a Qiagen RNeasy kit (Valencia, CA). TaqMan gene expression primers and probes for CHK1 (Hs00967506_m1) and GAPDH (Hs99999905_m1) were purchased from Applied Biosystems (Carlsbad, CA). GAPDH was used as an endogenous control and gene expression relative quantity was calculated using the ΔΔC_t_ method. Gene expression assays were performed in triplicates on an ABI StepOnePlus Real-Time PCR system.

### Small molecular inhibitors of CHK1

The small molecular CHK1 inhibitor AZD7762 [26] was purchased from Axon Medchem (Groninberg, Netherlands) and CHK1 inhibitor PF477736 [27] was purchased through SelleckChem. The drugs were reconstituted in dimethyl sulfoxide (DMSO) and aliquots were stored at –20 °C. An equivalent amount of DMSO for the highest concentration of drugs were used as a vehicle-control for each experiment.

### Western blotting

RIPA buffer (Thermo Scientific, Rockford, IL) in addition with protease inhibitor was used to obtain protein lysates from samples. Western blotting was performed on medulloblastoma cells treated with AZD7762 and PF477736 using standard methods. Antibodies for phosphorylated-ATM (S1981, Cat. No. 13050), phosphorylated-ATR (S428, Cat. No. 2853), CHK1 (2G1D2, Cat. No. 2360), phosphorylated-CHK1 (S345, Cat. No. 2341), CHK2 (Cat No. 2662S), phosphorylated-p53 (S15, Cat. No. 9286), phosphorylated-H2AX (S139, Cat. No. 9178), and α-tubulin (11H10, Cat. No. 2125) were purchased from Cell Signaling Technology (Danvers, MA), phosphorylated-RPA32 (S4/S8, Cat. No. A300-245A) was purchased through Bethyl Laboratories, Inc. (Montgomery, TX), phosphorylated-CHK2 (T68, Cat. No. 16297) was purchased through Santa Cruz Biotechnology, Inc. (Dallas, TX), anti-p53 (EPR17343, Cat. No. 179477) was purchased through Abcam, plc. (Cambridge, MA), and Actin (MAB1501) was purchased from Millipore. All primaries diluted to a 1:1,000 concentration, with the exception of actin which was diluted to 1:10,000, in 5% (w/v) milk in TBST, phosphorylated-CHK2 which was diluted to 1:250, in 5% BSA in TBST, phosphorylated-p53 which was diluted to 1:500, in 5% BSA in TBST, and p53 which was diluted to 1:2,000, in 5% milk in TBST. Secondary antibodies conjugated to horseradish-peroxidase were used in conjunction with chemiluminescent reagent to visualize protein bands.

### Colony formation and methylcellulose assay

In a 6-well plate, 500 cells were seeded in triplicate for 24 hours. Following seeding, cells were treated with 20 nM AZD7762, 175 nM cisplatin, or combination of both for 72 hours. At the end of treatment period, drug was removed and cells were allowed to grow in normal culture media. After seven days following seeding, culture media was aspirated. Cells were washed with PBS and colonies were stained with 0.5% crystal violet/25% methanol solution. Colonies were imaged using a GelCount™ (Abingdon, United Kingdom) imager, and colonies composed of ≥50 cells were counted.

For methylcellulose assay, D283 cell line (medulloblastoma suspension cell line) was seeded at a density of 1,000 cells per well in a 1.3 % methylcellulose solution containing AZD7762 at variable concentration. Following seeding, cells were allowed to form colonies for a period of 10 days. At the end of incubation period, colonies were counted using nitro blue tetrazolium (NBT) (1mg/mL in 1X PBS) and counted using GelCount™ (Abingdon, United Kingdom). Relative colony numbers were subsequently determined by normalizing colony counts against the average vehicle treated control colony count.

### Cell proliferation and viability assays

Cell proliferation was determined, in one method, by MTS [3-(4, 5-dimethylthiazol-2-yl)-5-(3-carboxymethoxyphenyl)-2-(4-sulfophenyl)-2H-tetrazolium] assay using CellTiter 96 Aqueous One Solution (Promega, Madison, WI). Cells were seeded for 24 hours before drug treatment. AZD7762 was added following seeding for a 72 hours at which point 30 μL of MTS reagent was added to the wells to make a final volume of 180 μL. The optical densities of wells were recorded using BioTek Synergy 2 plate reader (Winooski, VT) every hour for 3 hours after addition of the reagent. Background absorbance was subtracted from all well before analysis and IC_50_ values for the drug were calculated from the corrected absorbance values using GraphPad Prism. For combination drug treatment, different concentrations of cisplatin were used. Synergy between AZD7762 and cisplatin was determined with MTS absorbance data using Chou-Talalay Combination Index Theorem (CIE) [41].

Additionally, real-time measurement of cell proliferation was performed using an xCelligence RTCA SP instrument (Acea Biosciences, San Diego, CA) housed in an incubator maintained at normal cell culture parameters. A background sweep was initially acquired before seeding cells and monitoring for 24 hours. At 24 hours, AZD7762 or PF477736 were overlayed onto Daoy cells. Cellular proliferation was monitored for up to 100-hours total run time. Daoy cells transfected with siCHK1 or siNegative control were seeded onto 96-well xCelligence plate, and real-time cell proliferation was measured.

To determine cell viability, cells were seeded for 24 hours prior to treatment with a range of CHK1 inhibitors, AZD7762 and PF477736, with concentrations encompassing the IC_50_ for 72-hours. The viable cell concentration was determined following staining with Guava ViaCount reagent (Millipore, Billerica, MA). Samples were run on a Guava EasyCyte Plus flow cytometer (Millipore).

### Immunofluorescence by flow cytometry

Cells were seeded in triplicates in a 6-well plate for 24 hours and treated with AZD7762 for a period of 72 hours. Following treatment, cells were washed with PBS and incubated in BD Cytofix™ fixation buffer (#554655) for 10 minutes. Samples were subsequently re-washed with PBS before incubation with 1:10 dilution of anti-γH2AX (Ser139) conjugated antibody in the dark for 1 hour. Cells were washed again with PBS and incubated for 5 minutes using 0.1% Triton™ X-100 to permeabilize them. Antibodies were removed, samples were washed in PBS and counter-stained using 1:1000 dilution of 7-AAD in PBS for 15 minutes prior to acquisition on Guava EasyCyte Plus flow cytometer (Millipore). Acquired data was analyzed using FlowJo software (Ashland, Oregon).

### Caspase 3/7 assay using IncuCyteZOOM

Cells were seeded at 1,500 cells per well in a 96-well plate (Costar, Corning, NY). Cells were cultured at 37 °C and 5% CO_2_ and monitored using an IncuCyteZOOM (Essen BioScience, Ann Arbor, MI). Images were captured at 4-hour intervals from four separate regions per well using a 10X objective. Apoptosis was detected using CellEvent Caspase-3/7 Green Detection Reagent (#C10423) purchased from Life Technologies (Grand Island, New York). Each experiment was done in triplicate and relative apoptosis were plotted by nuclear cell count measurements.

### Statistical analysis

Prism 5 (GraphPad) was used to calculate IC_50_ values. Statistical significance was determined using a Student's t-test. Error bars represent the standard deviation (n*=*3).

## SUPPLEMENTARY MATERIALS FIGURES


