# Supplementary material for: Checkpoint kinase 1 expression is an adverse prognostic marker and therapeutic target in MYC-driven medulloblastoma
Source: Oncotarget. 2016 Jul 19;7(33):53881–94. doi: 10.18632/oncotarget.10692 (PMC5288228; doi:10.18632/oncotarget.10692)
Supplement: Supplementary file 1 [file oncotarget-07-53881-s001.pdf]

Checkpoint kinase 1 expression is an adverse prognostic marker and therapeutic target in MYC-driven medulloblastoma

SUPPLEMENTARY FIGURES

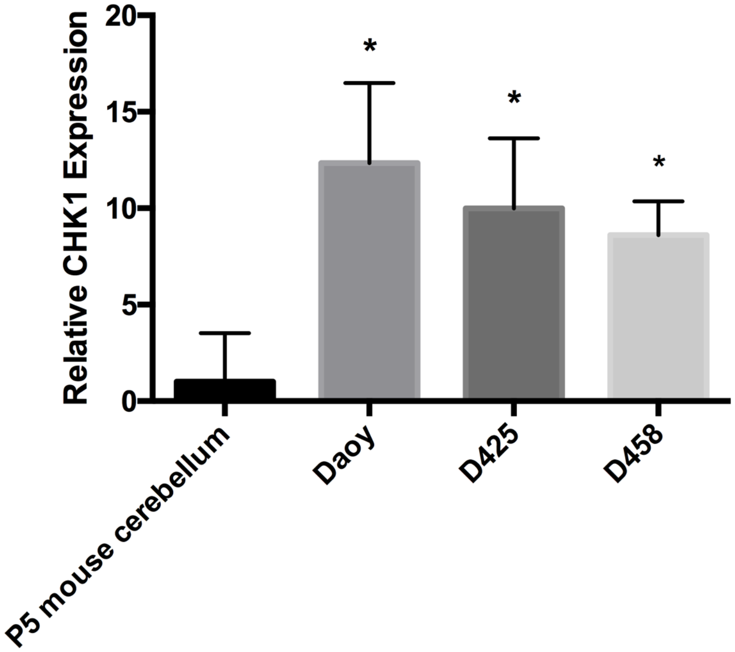

Supplementary Figure S1: CHK1 expression is lower in proliferating murine cerebellum compared to Group 3 medulloblastoma cell line as measured by qRT-PCR.

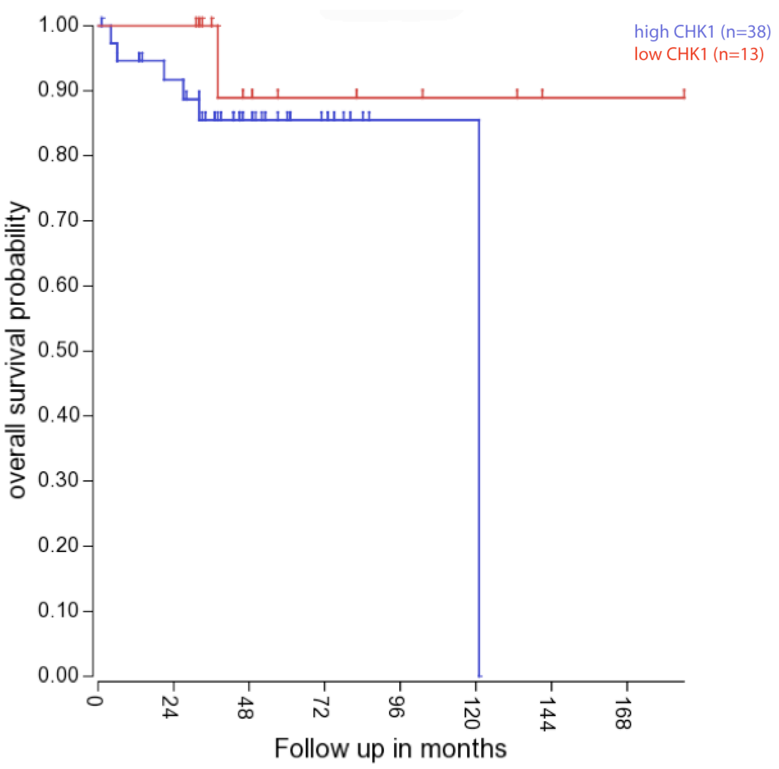

Supplementary Figure S2: CHK1 expression is not correlated with adverse outcomes in Group 4/SHH medulloblastoma patients. Kaplan-Meyer survival curve shows no significant change in long-term survival of group 4/SHH medulloblastoma patients with increased CHK1 expression.

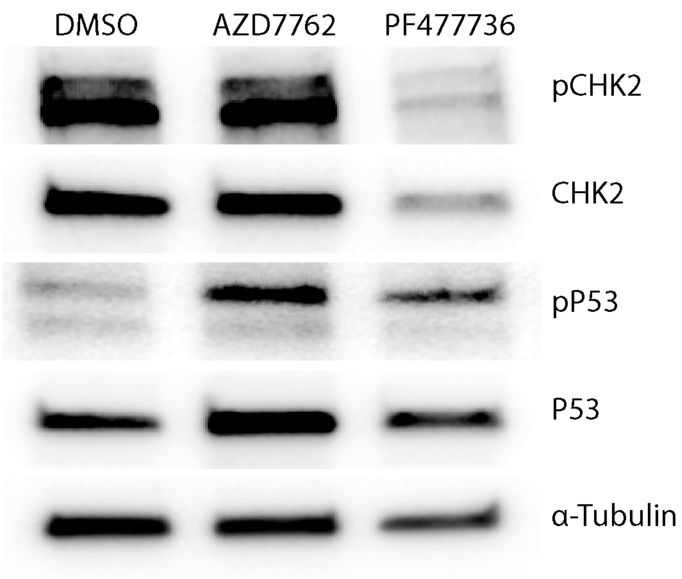

Supplementary Figure S3: Western blot of CHK2 and p53 phosphorylation in response to two different CHK1 inhibitors. AZD7761 suppresses phosphoCHK1 but not pCHK2 while PF477736 suppresses phosphorylation of both CHK1 and CHK2. Both inhibitors induce phosphorylation of p53.

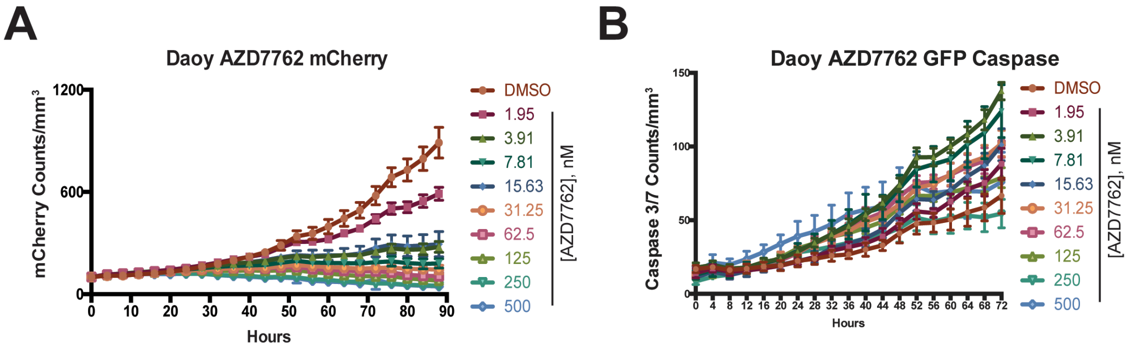

Supplementary Figure S4: AZD7762 reduces cell proliferation capacity and increases apoptotic rate. A. Real-time mCherry signal of Daoy cells transfected with mCherry after AZD7762 treatment. **B.** Real-time GFP Caspase-3/7 signal of Daoy cells treated with AZD7762.
